# Snow cover phenology dataset over global mountain regions from 2000 to 2023

**DOI:** 10.1016/j.dib.2024.110860

**Published:** 2024-08-22

**Authors:** Claudia Notarnicola

**Affiliations:** Eurac Research, Institute for Earth Observation, Viale Druso 1, 39100 Bolzano, Italy

**Keywords:** Snow phenology, MODIS, Mountain ecosystem, Water resources, Remote sensing, Climate change

## Abstract

Monitoring temporal and spatial changes in the mountain seasonal snowpack is a key step to better quantify the impact of recent climate warming on this very sensitive environment. In this context, to support the research on large scale, there is an urgent need of consistent and accurate data sets over global mountain regions. This paper presents a dataset derived from Moderate Resolution Imaging Spectroradiometer (MODIS) imagery collection 6.1 onboard of Terra satellite containing information on snow cover area and snow cover phenology (duration, start and end of the season) at 500 m ground resolution and covering the global mountain regions as delineated by the Global Mountain Biodiversity Assessment (GMBA) shapefile. The dataset contains yearly averaged snow cover area (SCA), snow cover duration (SCD), first snow day (FSD) and last snow day (LSD). The snow cover area SCA is based on snow cover fraction daily maps averaged on a yearly basis. To derive the snow phenology variables, SCD, FSD, LSD, a second-order autoregressive approach was adopted to interpolate the data thus reducing the impact of cloud coverage. The dataset was processed for the entire MODIS time series starting from October 2000 up to September 2023 and it is provided on yearly basis considering the hydrological year timeframe: from 1^st^ October to 30^th^ September of the subsequent year for the Northern Hemisphere and from 1^st^ April to 31^st^ March of the subsequent year for the Southern Hemisphere. The use of the data can be manyfold from quantifying the snowpack changes in the last 23 years, to addressing the impact of snowpack changes on water resources and vegetation phenology.

Specifications Table

**Table:** 

Subject	Environmental Sciences
Specific subject area	Global and Planetary Change, remote sensing, cryosphere
Type of data	Image
Data collection	This snow cover phenology dataset was generated by exploiting the snow product MOD10A1.061 (MODIS/Terra Snow Cover Daily L3 Global 500 m Grid). This product contains information on the Normalized Snow Differential Index (NDSI). The images were processed to obtain the following variables: yearly averaged snow cover area (SCA), snow cover duration (SCD), first snow day (FSD), last snow day (LSD). SCA maps were obtained by averaging at pixel level the NDSI values and transforming them in fractional snow cover through a linear relationship. SCD, FSD and LSD were obtained through an interpolated NDSI time series to cope with cloud obstruction. Processing was implemented in Google Earth Engine platform.
Data source location	The spatial coverage of the snow phenology dataset is: 180°W-180°E, 57°S-80°N over the global mountain regions as delineated by the reference layer Global Mountain Biodiversity Assessment v1.1 (GMBA) [1,2]. The MODIS MOD10A1.061 product can be downloaded from: https://nsidc.org/data/mod10a1 These data are also available on the GEE platform https://earthengine.google.com
Data accessibility	Repository name: Zenodo https://doi.org/10.48784/1zvv-nw59 Direct URL to data: https://zenodo.org/records/11181638
Related research article	C. Notarnicola, Hotspots of snow cover changes in global mountain regions over 2000–2018, Remote Sen Environ, 243 (2020), 111781, ISSN 0034-4257, https://doi.org/10.1016/j.rse.2020.111781.

## 1 Value of the Data

• The dataset presented in this paper can be used to monitor the seasonal snow dynamics over the last 23 years in global mountain areas [1]. Having a global dataset will also allow comparison across different mountain regions thus identifying similar and/or contrasting patterns and trends.
• The dataset can be used jointly with other information such as streamflow variables to exploit the nexus between seasonal snow cover changes and the related water resources in downstream areas.
• The spatial and temporal dynamics can be related to vegetation changes to address the influence of snow dynamics on the plant phenology.
• Even though the period of 23 years can limit the attribution of long-term trends, the dataset can be used to calibrate modelling data thus allowing to extend the time series to a longer period [3].

## 2 Background

Mountains play a special role being the water tower of the downstream areas and at the same time strongly affected by the recent climate warming [[4], [5], [6]]. In this context, it is crucial the assessment of snowpack changes that has a first direct influence on water resources availability with cascading impact on sectors such as agriculture, hydropower production, domestic water use, tourism.

Having consistent and accurate data on snow related variables is of utmost importance to allow an accurate quantification of the spatial-temporal changes. As pointed out by Bormann et al. [7] MODIS sensor offers an unprecedent possibility in term of time availability from 2000 to present and ground resolution (500 m) to monitor consistently mountain areas. The presented dataset fully exploits the MODIS time series to derive a set of variables to be used to assess the snow dynamics of the last 23 years [8]. With respect to other datasets [9], the dataset has the ground resolution of 500 m. In [1] the dataset was partially exploited to derive the snowpack changes over 2000–2018 by addressing the variability according to different altitudinal transects and, in relation to temperature and precipitation as main drivers of the snow dynamics. The results allowed quantifying the rate of changes of the snowpack across the globe.

## 3 Data Description

**Fig. 1:**
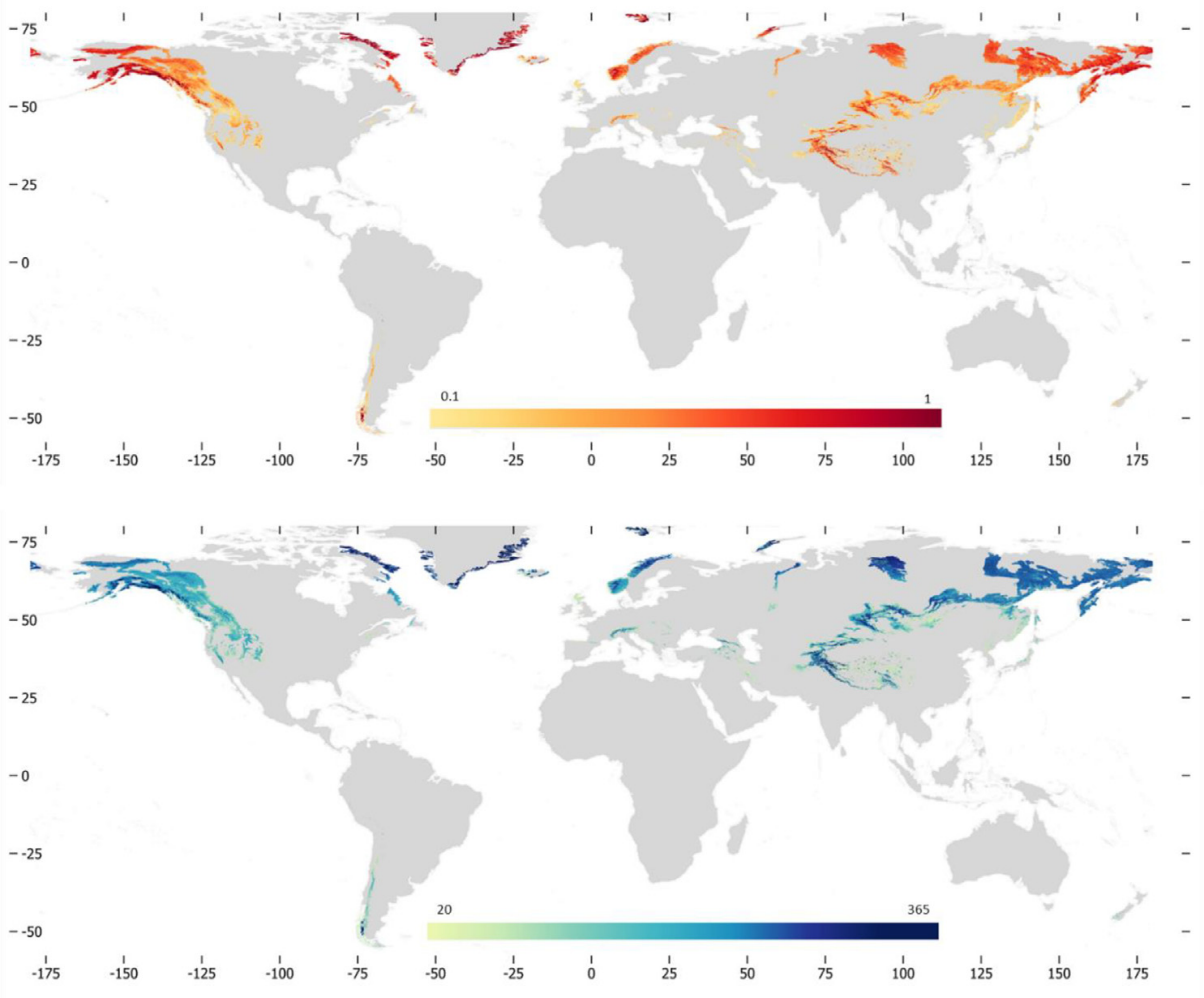
Examples of SCA (top) and SCD (bottom) maps for the year 2022–2023.

The main characteristics of the dataset are illustrated in Table 1 while Fig. 1 illustrates examples of maps for SCA and SCD for the year 2022–2023.

**Table 1:** Main characteristics of the dataset.

Geographical coverage	180°W-180°E, 57°S-80°N over the GMBA mountain regions shapefile
Spatial resolution	500 m
Temporal extent	Oct. 2000 to Sept. 2023
Temporal resolution	yearly
Spatial coordinate system	EPSG: 4326, WGS 84 geographic coordinate system

The variables derived from the MODIS Terra collection 6.1 product MOD10A1 are the yearly snow cover area (SCA), the snow cover duration in days (SCD), the first snow day (FSD) and the last snow day (LSD). They are contained in the folder “SnowCoverPhenology.zip”, in subfolders with the respective names of the variable (SCA, SCD, FSD, LSD). Table 2 reports the characteristics of the different variables.

**Table 2:** Characteristics of the different variables.

Snow variable	Definition	Value domain
Snow cover area (SCA)	Percentage of snow coverage at pixel level	1001–10,000 (actual value 0.1001 to 1)
Snow cover duration (SCD)	The number of days a pixel is covered by snow in the hydrological year	21–365 (days) (366 for leap years)
First snow day (FSD)	First date in the hydrological year that a pixel is snow covered	1–365 (days) (366 for leap years)
Last snow day (LSD)	Last date in the hydrological year that a pixel is snow covered	1–365 (days) (366 for leap years)

The calculation of the variables was carried out at yearly basis considering the hydrological year, that is from 1^st^ October to 30^th^ September of the subsequent year for the Northern Hemisphere and from 1^st^ April to 31^st^ March of the subsequent year for the Southern Hemisphere. The year associated to the variable file refers to the starting year, for example SCD2000 indicates the snow cover duration variable for the period 1^st^ Oct. 2000 to 30^th^ Sept. 2001 for the Northen Hemisphere and from 1^st^ April 2000 to 31^st^ March 2001 for the Southern Hemisphere. Due to their size, as the products are covering global mountain regions, they are divided into tiles. SCA and SCD are divided into three tiles with the following coordinates:

VariableNameYear-1 LAT/LON UL 80°N, 180°W, LR 57°S, 32° 50‘W
VariableNameYear-2: LAT/LON UL 80°N, 32° 50‘W, LR 57°S, 114°21‘E
VariableNameYear-3 LAT/LON UL 80°N, 114°21′E, LR 57°S, 180° E

While for FSD and LSD into two tiles with the following coordinates:

VariableNameYear-1: LAT/LON UL 80°N, 180°W, LR 57°S, 30° E
VariableNameYear-2: LAT/LON UL 80°N, 30°E, LR 57°S, 180° E

In the case of SCA variable, the single files report the following name “SCAint_Year” as the data were rescaled to reduce the file size (scale factor = 10,000).

To make comparison among different years, for FSD and LSD, it can be advisable to rescale the data, that is to subtract 365 (366 for the leap years) for the first part of the hydrological year. In this way, the dates between October to December (North Hemisphere), April to December (South Hemisphere) will be negative, while the remaining ones positive. This will allow the users to easily distinguish the dates across the two calendar years.

To remove the ephemeral snow that can affect the different areas and to consider the main seasonal snow cover and phenology patterns excluding pixels with reduced snow coverage, the dataset was filtered thus retaining only the pixels where both SCA>10 % and SCD > 20 days. The threshold on SCD is derived from the validation results as indicated in Table 3 where the mean absolute error (MAE) in the comparison with ground data is around 20 days. For SCA, the threshold is consistent with the mean value of the errors derived from the validation of the snow cover fraction approach [10].The masks derived from these thresholds were applied to FSD and LSD so to have a consistent data set in each year. The masked values are set to 0. The volume of the dataset is 8.41 GB (6.54 GB in compressed form).

## 4 Experimental Design, Materials and Methods

**Fig. 2:**
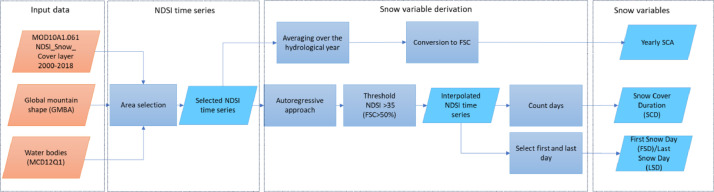
Overall workflow for the snow cover phenology variables processing. The same procedure is repeated separately for the Northern and Southern Hemisphere.

The approach for the derivation of the snow variables is presented in Fig. 2. For the whole processing, the cloud platform Google Earth Engine (GEE) was adopted for easiness to access the full

MODIS time series and the processing capability of such large dataset [11]. The approach was applied to global mountain regions as delineated by the reference layer Global Mountain Biodiversity Assessment (GMBA) [1]. The main input data is the MOD10A1.061, from which the Normalized Difference Snow Index (NDSI) layer (NDSI_Snow_Cover) was considered. Only MODIS Terra data were exploited due to the malfunctioning of some detectors in MODIS Aqua which determines a reduced accuracy in the detection of snow cover [12]. Other input data is the MODIS Land Cover Type product MCD12Q1 used to mask out the water bodies. Considering the different time frames of the hydrological year for the Northern and Southern Hemisphere, all the processing steps were done separately for the two hemispheres and then the two parts were mosaicked to obtain the final product.

### 4.1 Yearly SCA derivation

The Snow Cover Area (SCA) is provided as a yearly reference to be compared with the snow phenology variables. It is derived from the NDSI_Snow_Cover layer of the MOD10A1.061 product with values in the range 0–100. This product already has masks for cloud, ocean and night. The daily acquisition over each hydrological year is averaged at pixel level and then is converted into fractional snow cover FSC (percentage of snow presence in each pixel) by considering the approach proposed by Salomonson and Appel [10] to obtain a yearly SCA value for each pixel. This approach was selected because it has showed good performances, and it is easy to implement at global scale. For more regional studies, other approaches need to be addressed in order to consider the complexity of the terrain [13]. As the main aim to produce an average value of SCA and the averaging procedure over one entire year contributes to reduce the influence of gaps due to cloudiness, for this variable no gap-filling approach was applied [1,14].

### 4.2 Snow phenology derivation

The snow phenology parameters (SCD, FSD, LSD) were derived from the daily NDSI maps. To obtain the SCD values which indicate for each pixel the number of snow-covered days, there was the need to fill the gaps due to the cloud presence as in this case the impact of cloud presence can be notable. To this aim, a second-order autoregression was considered, where the value at time t is derived as a linear relationship from the values at time t-1 and t-2, with clear sky conditions [14,15]. The approach was chosen as compromise between computation complexity and capability to reduce cloud coverage. When no information on t-1 and t-2 days are available, the last available value is propagated until the next cloud free image is available. Notwithstanding the simplicity, the approach presents robust results when compared with both ground data and model simulations. Indeed, the validation results were checked against the percentage of missed pixels (due to cloud cover and polar darkness) thus indicating only a slight worsening of the results in the case with more than 70 % of missing values [1]. In the counting step to derive the number of days of snow presence in each pixel, to reduce the uncertainties related to low values of FSC and patchy snow presence, only pixel with FSC > 50 % (corresponding to NDSI > 35) were considered as snow covered and set to 1, while the pixels where the FSC < 50 % were set to 0 [1,15]. For the selection of the threshold on FSC values, a sensitivity analysis was carried out. The threshold was selected among the following ones: 13 %, 25 %, 50 %, 75 % and was based on a comparison of satellite-based SCD, FSD and LSD values with the corresponding ones derived from ground data. The comparison indicates that the threshold 50 % provides the most robust results considering different performance metrics [1].

FSD and LSD represent the first and the last date in the hydrological year with snow presence. The FSD and LSD have been revised because of the intense processing and in view of the validation results [1]. In a preliminary version, their calculation was based on the NDSI interpolated values and adopted a moving window of 15 days to identify the first or the last period of 5 days of continuous snow [16,17].

After the validation, the error associated to the retrieval of the FSD and LSD was found close to the moving window selected, and moreover it was found that the posed conditions of FSC>50 %, already reduces the detection of ephemeral snow related to short snowfall events at the beginning and end of the season. For these reasons, FSD and LSD were detected as the first and the last day with snow appearance respectively. However, when detecting LSD, there is still the possibility that the first snow of the subsequent year can be detected, especially at high latitude locations. To avoid this possible mistake, the LSD search was done considering interval between 15 days and one month starting from end of July for Northern Hemisphere and end of January for Southern Hemisphere. In this case, LSD was considered as the first snow disappearing in the time series.

### 4.3 Collection 6.0 and 6.1

It is worthwhile mentioning that the work in [1] was conducted over the MODIS collection 6, while the dataset presented in this paper was derived from the products MOD10A1 collection 6.1 which fully substituted the previous collection. The minor changes introduced in the new collection can bring some differences that will only marginally affect the snow and ice products. The two collections are considered largely compatible by the producers [18].

### 4.4 Validation

The snow phenology variables were validated as shown in Notarnicola 2020 [1]. For the validation of the SCD, FSD and LSD parameters, time series of ground snow depth were exploited. The data were selected so to have the most consistent time series with reduced gaps, to avoid interpolation which may introduce further uncertainty. The data were distributed worldwide deriving from USA, Canada, Argentina, Italy, France, Norway, China, Russia for a total of 466 time series. The selection of the ground data took into consideration different elevation belts and land cover types. Here in this work the validation with ground data was repeated to verify possible differences between collection 6 and 6.1 that may have influenced the whole processing and the final products. Table 3 summarizes the main results of the validation procedure for both collection 6 and 6.1.

**Table 3:** Main figures of merit in the comparison between snow phenology products and ground data. ** indicates a 5 % significance level.

	SCD			FSD			LSD		
	R	MAE	Bias	R	MAE	Bias	R	MAE	Bias
C6	0.84**	21.1	−3.1	0.93**	11.1	4.7	0.89**	13.9	−2.2
C6.1	0.84**	21.4	−3.0	0.93**	11.4	5.4	0.88**	14.4	−3.0

## Limitations

Glaciers are not masked and there no specific processing carried out for glacier surface.

The water bodies are masked by using the MODIS products MCD12Q1. In some cases, there could be still some commission errors of snow detection on the borders of lakes. If applying these data on a specific region, the use of a more detailed and accurate local lake maps is advisable.

In the area of South America (area defined by the following coordinates UL 75°W, 45°S, LR 72°W, 52°S), some spatial patterns appear, especially evident over glacier areas. They are related to some issues in the MOD35_L2 product which is used to mask clouds in the snow product MOD10A1. The gaps in the cloud mask can be related to cases when the input radiances are of poor quality or incomplete. If the cloud mask is missing or wrongly delineate, it can lead to wrong identification of snow presence (e.g. cloud detected as snow) [19]. Occasionally patterns related to wrong detection of cloud mask may be found in other regions.

As the dataset was filtered for SCA>10 % and SCD>20 days, this dataset can have some limitations to analyze regions characterized by snow presence in more intermittent way [20].

## Ethics Statement

The author confirms that the current work does not involve human subjects, animal experiments, or any data collected from social media platforms.

## CRediT Author Statement

Claudia Notarnicola: Conceptualization, Methodology, Software, Validation, Resources, Data curation, Visualization, Writing-original draft.

## Data Availability

Global mountain snow cover phenology from MODIS/Terra imagery (Original data) (Zenodo).
